# A very-low-calorie ketogenic diet normalises obesity-related enhanced levels of erythropoietin compared with a low-calorie diet or bariatric surgery

**DOI:** 10.1007/s40618-024-02364-9

**Published:** 2024-05-02

**Authors:** A. Fernandez-Pombo, P. M. Lorenzo, M. C. Carreira, D. Gomez-Arbelaez, A. I. Castro, D. Primo, J. Rodriguez, I. Sajoux, J. Baltar, D. de Luis, D. Bellido, A. B. Crujeiras, F. F. Casanueva

**Affiliations:** 1 Epigenomics in Endocrinology and Nutrition Group, Epigenomics Unit, Instituto de Investigacion Sanitaria de Santiago de Compostela (IDIS), Complejo Hospitalario Universitario de Santiago de Compostela (CHUS/SERGAS), Travesía da Choupana Street s/n, 15706 Santiago de Compostela, La Coruna Spain; 2 Endocrinology and Nutrition Division, Complejo Hospitalario Universitario de Santiago de Compostela (CHUS/SERGAS), Santiago de Compostela, Spain; 3 CIBER Fisiopatologia de La Obesidad y Nutricion (CIBERobn), Madrid, Spain; 4 Molecular Endocrinology Group, Instituto de Investigacion Sanitaria de Santiago de Com-Postela (IDIS), Complejo Hospitalario Universitario de Santiago de Compostela (CHUS) and Santiago de Compostela University (USC), Santiago de Compostela, Spain; 5 Faculty of Health Sciences, University of Santander (UDES), Bucaramanga, Colombia; 6 Center of Investigation of Endocrinology and Nutrition, Medicine School and Department of Endocrinology and Investigation, Hospital Clinico Universitario, University of Valladolid, Valladolid, Spain; 7 Clinical Biochemistry Laboratory, Complejo Hospitalario Universitario de Santiago de Compostela (CHUS/SERGAS), Santiago de Compostela, Spain; 8 Medical Department Pronokal Group, Barcelona, Spain; 9 Division of General Surgery, Complejo Hospitalario Universitario de Santiago (CHUS/SERGAS), Santiago de Compostela, Spain; 10 Endocrinology and Nutrition Unit, Complejo Hospitalario Universitario de Ferrol (CHUF/SERGAS), Ferrol, Spain

**Keywords:** Adiposity, Erythropoietin, Nutritional ketosis, Energy restriction, Ketone bodies

## Abstract

**Purpose:**

Nutritional ketosis synergistically with body-weight loss induced by a very-low-calorie ketogenic diet (VLCKD) has proven to be effective in improving obesity-related pathophysiology. Recently, growing attention has been focused on the relation between erythropoietin (EPO) and obesity. Thus, this study aims to investigate whether nutritional ketosis and weight loss induced by a VLCKD modify the circulating levels of EPO in patients with obesity in comparison with the effect of low-calorie diet (LCD) or bariatric surgery (BS).

**Methods:**

EPO levels, iron status and body composition parameters were evaluated in 72 patients with overweight or obesity and 27 normal-weight subjects at baseline and after the three different weight-reduction therapies (VLCKD, LCD and BS) in 69 patients with excess body weight. β-hydroxybutyrate levels were also measured in the VLCKD group. The follow-up was established at 2–3 months and 4–6 months.

**Results:**

It was found that EPO levels were higher in morbid obesity and correlated with higher basal weight, fat mass (FM) and fat-free mass (FFM) in the overall sample. High baseline EPO levels were also correlated with higher impact on the course of weight loss and changes in FM and FFM induced by the three weight-loss interventions. Furthermore, the VLCKD induced a decrease in EPO levels coinciding with maximum ketosis, which was maintained over time, while statistically significant changes were not observed after LCD and BS.

**Conclusion:**

The obesity-related increased EPO levels are restored after VLCKD intervention at the time of maximum ketosis, suggesting a potential role of the nutritional ketosis induced by the VLCKD. Baseline EPO levels could be a biomarker of response to a weight-loss therapy.

**Graphical abstract:**

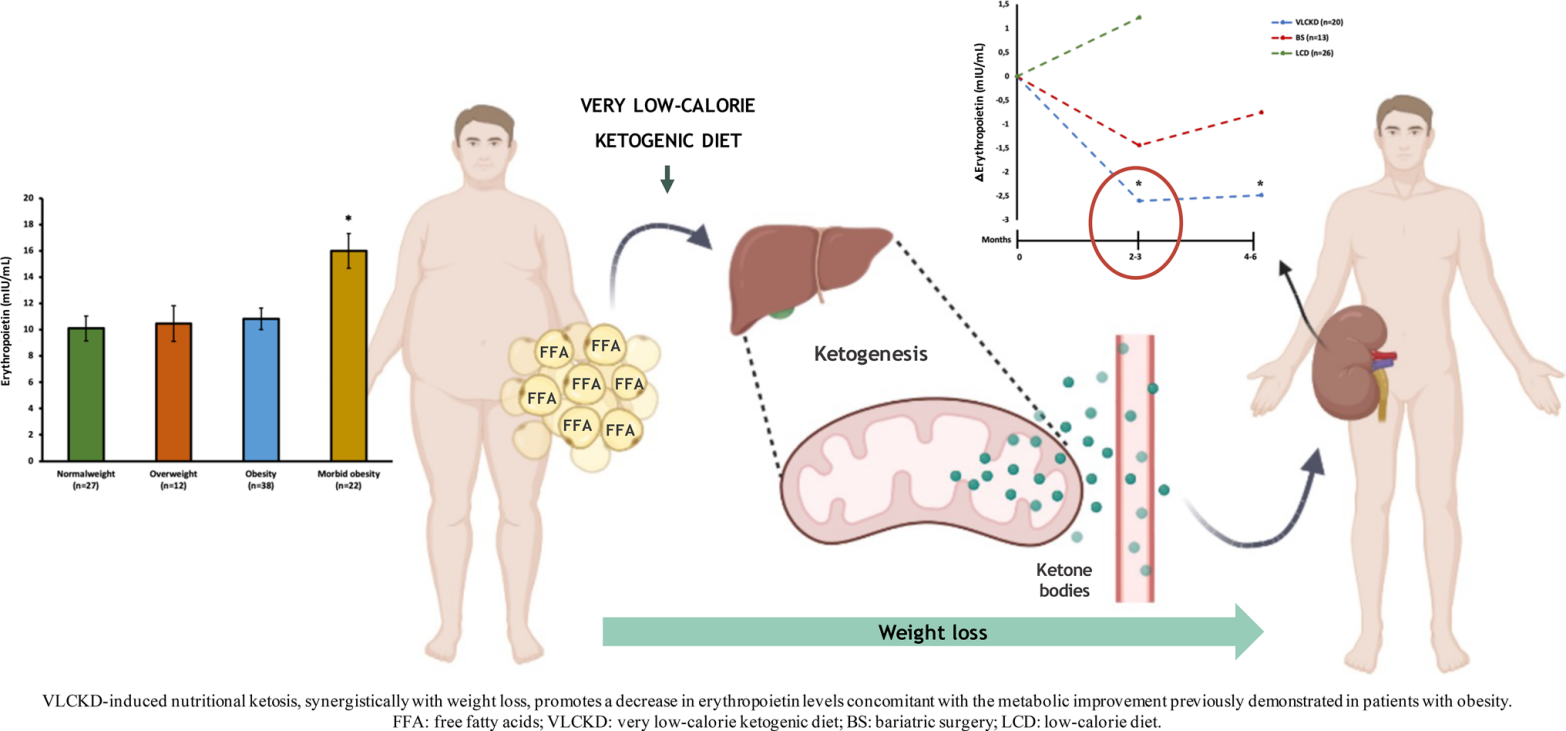

## Introduction

Obesity is considered to be a persistent worldwide health epidemic. Despite advances in its management, the incidence of this disorder and its complications is still rising, suggesting that more attention should be focused on developing new therapies [1, 2]. Identifying possible regulatory factors in energy homeostasis, adiposity, and changes in body weight is, therefore, essential to provide new preventive and therapeutic strategies for obesity and its associated metabolic syndromes.

Erythropoietin (EPO) is a glycoprotein hormone, primarily secreted in the kidney, which is necessary for erythrocyte production [3, 4]. It has classically been used for the treatment of anaemia in chronic kidney disease, including patients with type 2 diabetes undergoing haemodialysis therapy [5, 6]. In addition to its indispensable role during erythropoiesis, its biological activity extends to non-hematopoietic effects related to the improvement of metabolic disorders [7].

Thus, in recent years, growing attention has been focused on the role of EPO in obesity. In animal model studies, mice with EPO receptor (EPOR) expression limited to hematopoietic tissues and mice with adipocyte-specific deletion of EPOR exhibited obesity and decreased insulin sensitivity and glucose tolerance [8–10]. In addition, treatment of diet-induced obese rodents with exogenous EPO reduced food intake and preadipocyte differentiation and increased fat oxidation, leading to improved fat mass (FM), glucose tolerance and insulin resistance [11–13]. In contrast, in humans, EPO was found to be increased in subjects with obesity and anaemia [14]. It has also been shown that EPO levels are higher in subjects with metabolic syndrome as well as in individuals with abdominal obesity component [15].

Among the potential mechanisms underlying the regulation of EPO synthesis and secretion, it has been proposed that ketone bodies could play a relevant role. Proof of this hypothesis could be the hyperketonaemia induced with sodium-glucose cotransporter 2 (SGLT2) inhibitor therapy concomitant with an increase in haematocrit [16], moderate weight loss [17] and increase EPO secretion by the kidney [18]. On the contrary, in a recent study, the supplementation of β-hydroxybutyrate (β-OHB) in patients with prediabetes did not affect iron metabolism [19]. On the other hand, it is also known that hypoxia is a stimulator of EPO production [3] and that humans living at high altitudes are less likely to be obese [20]. However, a decrease in EPO levels has also been linked to continued exposure to hypoxia at high altitude due to adaptative responses [21]. Thus, the specific role of EPO in the management of human obesity is yet to be elucidated. We hypothesised that the treatment of obesity with a very-low-calorie ketogenic diet (VLCKD) could reduce the enhanced levels of EPO associated to obesity via the action of nutritional ketosis synergistically with its effect on body composition [22] and muscle function [23], as well as the improvement in inflammatory and oxidative stress markers [24]. The choice of a VLCKD is justified by its previously demonstrated efficacy with associated improvement in metabolic and cardiovascular parameters, not only by group experience but also by scientific evidence, which is why it has been included in recent clinical practice guidelines in the treatment of obesity and diabetes [25, 26]. The clinical difference is clear with preservation of muscle mass and better patient adherence.

This study aimed to investigate whether the nutritional ketosis and weight loss induced by a VLCKD lead to changes in EPO circulating concentration, compared with a standard, balanced low-calorie diet (LCD) or bariatric surgery in patients with obesity.

## Materials and methods

### Study population

A total of 99 subjects were enrolled in this study, of whom 72 were overweight or had obesity and 27 were healthy, normal-weight volunteers (control group). Of the 72 patients with overweight or obesity, 69 completed a weight-reduction therapy based on one of two different energy-restriction programmes or bariatric surgery. Thus, one group of subjects received a VLCKD (Pronokal method®), another group followed an LCD, and the third group underwent bariatric surgery.

The inclusion criteria were to be between 18 and 65 years of age, body mass index (BMI) ≥ 30 kg/m^2^, stable body weight in the previous 3 months, a desire to lose weight and a history of failed dietary efforts. The main exclusion criteria were diabetes mellitus, obesity induced by other endocrine disorders or by drugs and participation in any active weight loss programme in the previous 3 months. In addition, those patients with previous bariatric surgery, known or suspected abuse of narcotics or alcohol, severe depression or any other psychiatric disease, severe hepatic insufficiency, any type of renal insufficiency or gout episodes, nephrolithiasis, neoplasia, previous events of cardiovascular or cerebrovascular disease, uncontrolled hypertension, orthostatic hypotension and hydroelectrolytic or electrocardiographic alterations were excluded. Females who were pregnant, breastfeeding, or intending to become pregnant, and those with child-bearing potential who were not using adequate contraceptive methods were also excluded. Apart from obesity and metabolic syndrome, participants were generally healthy individuals.

## Study design

### Very-low-calorie ketogenic diet

A cohort of 20 patients with obesity (12 women, mean age 47.2 ± 10.2 years, mean body mass index [BMI] 35.6 ± 4.4 kg/m^2^), attending the Obesity Unit at the University Clinical Hospital of Santiago, Spain, was selected to receive a VLCKD. This VLCKD was designed according to a commercial weight-loss programme (Pronokal method ®), which includes lifestyle and behavioural modification support as previously described [22, 27–29]. This method is based on high-biological-value protein preparations obtained from cow’s milk, soya, avian eggs, green peas and cereals. Each preparation contains 15 g protein, 4 g carbohydrates, 3 g fat and 50 mg docosahexaenoic acid, and provides an energy intake of 90 to 100 kcal [22].

The weight-loss programme had three phases. The first phase consisted of a VLCKD (600–800 kcal/day), low in carbohydrates (< 50 g/day from vegetables) and lipids (10 g/day from olive oil), with a protein intake corresponding to 0.8–1.2 g/kg of ideal body weight. Throughout this ketogenic phase, supplements of vitamins and minerals such as K, Na, Mg, Ca and omega-3 fatty acids were provided. When the weight-loss target was achieved, the ketogenic phase ended, and patients started a low-calorie diet (800–1500 kcal/day) followed by a maintenance diet of 1500–2000 kcal/day. The weight-loss programme had five steps [22] and adhered to the 2015 guidelines of the European Food Safety Authority regarding total carbohydrate intake [30].

Patients followed the different steps of the programme for a maximum period of 4–6 months, although they remained under medical supervision for the following 12 months [22, 27–29]. The intervention included an evaluation by the specialist physician conducting the study and an assessment by an expert dietician. All patients also underwent a structured programme of physical exercise, with external supervision [22].

Throughout the study the patients completed a maximum of ten visits with the research team (every 15 ± 2 days), of which four visits were for the performance of a complete physical, anthropometric and biochemical assessment, while the remaining visits aimed to control adherence and evaluate potential side effects. The four assessment visits were scheduled according to the development of ketosis for each patient as follows: normal level of ketone bodies (baseline), maximum ketosis (2–3 months), reduction of the ketotic approach due to partial reintroduction of normal nutrition (around 3 months) and no ketosis (4–6 months, end of the study) [22]. In all the visits, patients received dietary instructions, individual supportive counselling and encouragement to exercise on a regular basis using a formal exercise programme. Anthropometric, body composition and biochemical data were evaluated at baseline (0 months), at maximum ketosis (2–3 months) and at no ketosis (4–6 months).

The total ketotic state lasted for 60–90 days. As previously described [22], ketosis was determined by measuring ketone bodies, specifically β-OHB in capillary blood using a portable meter (GlucoMen LX Sensor, A. Menarini Diagnostics, Neuss, Germany) before measurements of anthropometric parameters. All of the determinations of capillary ketonemia were made after overnight fasting of 8 to 10 h. These measurements were performed daily by each patient during the entire VLCKD and the corresponding values were reviewed on the machine’s memory by the research team for managing adherence. Additionally, β-OHB levels were determined at each complete visit by the physician in charge of the patient. The measurements reported as “low value” (≤ 0.2 mmol/L) by the meter were assumed to be zero for the purposes of statistical analyses.

### Low-calorie diet

A cohort of 26 patients with obesity (13 women, mean age 43.2 ± 14.6 years, mean BMI 33.3 ± 3.7 kg/m^2^) followed a therapy programme based on a nutritional intervention carried out by trained dieticians from the Department of Endocrinology and Nutrition of Valladolid Clinical Hospital. During this interventional study (12 weeks), the subjects received individualised counselling on a hypocaloric diet with a Mediterranean profile. This caloric intake was calculated by subtracting 500 kcal from the caloric intake obtained with the Harris Benedict formula in our population with obesity. The ratio of nutrients in the diet was as follows: 25% from lipids, 23% from proteins and 52% from carbohydrates. The range of fats was: 50.7% of monounsaturated dietary fats, 38.5% of saturated dietary fats and 11.8% of polyunsaturated dietary fats. The diet contained the following foods: extra-virgin olive oil (30 ml/day), 3 portions of fish per week, 3 portions of nuts per week, 4–5 portions of vegetables and fresh fruit each day. The monitoring of the dietary intervention was carried out each 2 weeks by a dietitian. All subjects received briefing to record their intakes for three different days. Moreover, all participants were asked to maintain their normal physical activity during the study. Anthropometric, body composition and biochemical data were evaluated at baseline (week 0) and at the end of the dietary intervention (endpoint, 3 months). This protocol is established on a standard hospital-based nutritional therapy to induce weight loss, with the protocol being performed over a two-month period, until a minimum of 5% reduction in weight is achieved.

### Bariatric surgery

A cohort of 23 patients with obesity (18 women, mean age 43.9 ± 9.66 years, mean BMI 45.5 ± 6.8 kg/m^2^) underwent bariatric surgery via laparoscopy at the University Clinical Hospital of Santiago, Spain. In the 2–4 weeks prior to surgery, the patients received a low-calorie diet providing approximately 900 kcal/day (119.6 g of carbohydrates [53%], 70.4 g of proteins [31%] and 15.5 g of lipids [15%]), which included 250 mL of Optisource Plus^□^, Nestlé HealthCare Nutrition, three times a day [31]. After the surgical intervention, the patients were advised to follow a special diet that consisted of 1 month of semi-liquid diet before progressively introducing solid foods until a diet of normal consistency was reached, providing about 850 kcal/day (110 g of carbohydrates [52%], 50 g of proteins [23%] and 23 g of lipids [24%]) [31]. The patients subjected to bariatric surgery were provided with a daily vitamin-mineral supplement, beginning on the day of the surgical procedure, to reduce the risk of developing nutritional deficiencies [32]. The monitoring of the dietary intervention was carried out monthly by a dietitian. In accordance with protocol, all the patients with morbid obesity and/or those proposed for bariatric surgery included in the current study were evaluated in the Department of Pneumology, with an arterial blood gases test being carried out in all cases to discard possible untreated hypoxemia. Anthropometric, body composition and biochemical data were evaluated at baseline (week 0), at 3 months (endpoint) and 6 months after bariatric surgery (follow-up). These data were available at baseline for the 23 patients evaluated and at the three visits for 13 patients.

## Anthropometry and body composition

Body weight and height measurements were performed using a wall-mounted stadiometer (Seca 220 scale, Medical Resources, EPI Inc., Birmingham, AL, USA). Total body composition was evaluated via bioelectrical impedance analysis (BIA) after 8–12 h of overnight fasting (InBody 720; Biospace, Tokyo, Japan).

## Biochemical analysis

Blood samples were taken between 8:00 and 9:00 a.m. after 12 h overnight fasting, and EDTA-plasma and serum were separated from whole blood and immediately frozen at −80 °C until assay. EPO and soluble transferrin receptor (sTfR) circulating levels were analysed using a commercial enzyme-linked immunosorbent assay (ELISA) (Siemens healthcare diagnostics, SL, Spain) according to the manufacturers’ instructions. Iron metabolism parameters were analysed following usual clinical practice of the central laboratory at our reference hospital. Circulating ketone bodies, specifically β-OHB, were measured in capillary blood using a portable meter (GlucoMen LX Sensor, A. Menarini Diagnostics, Neuss, Germany).

## Statistical analysis

The sample size of the current study was calculated to detect differences in EPO and sTfR levels considering published values and standard deviations [15] and was calculated to achieve an α = 0.05 and a power (1-β) of 80%. The normal distribution of variables was explored using the Kolmogorov–Smirnov and Shapiro–Wilk tests. Analysis of variance (ANOVA) was used to study differences between groups. Moreover, the concentration of EPO was correlated with age (data not shown) and, therefore, the analysis of EPO concentrations was adjusted by age in an analysis of covariance (ANCOVA). The repeated-measures ANCOVA test was used to study the effects of the time-course of weight-loss therapies and groupings of body composition, as well as EPO and sTfR levels in patients with obesity, adjusted by age and baseline BMI. Differences with respect to baseline (0 months) were evaluated by the paired Student’s *t*-test within each weight-loss treatment. Differences in the changes respect to baseline between weight-loss treatments were evaluated with ANCOVA adjusted by gender and baseline BMI. Men and women were analysed together. The potential association between EPO levels and sTfR levels was evaluated using the Spearman coefficient test. Statistical analyses were performed for the data from patients who had valid data for all three time-points of the treatment follow-up (1: baseline, 2: follow-up, 3: endpoint of the treatment). Statistical analyses were performed using SPSS version 22.0 software (SPSS Inc., Chicago, IL, USA) for Windows XP (Microsoft, Redmond, WA, USA). A *p* value ≤ 0.05 was considered statistically significant.

## Results

A total of 99 patients were evaluated. The cohort of patients with excess body weight was classified according to their BMI into the following categories: overweight range (25–29.9 kg/m^2^, *n *= 12), obesity (30–39.9 kg/m^2^; *n* = 38) and morbid obesity (≥ 40 kg/m^2^; *n* = 22) and compared with a group of normal-weight (BMI 18.5–24.9 kg/m^2^; *n* = 27) individuals. The demographic data, along with analytical, anthropometric and body composition data at baseline of these subjects in terms of adiposity are shown in Table 1.

**Table 1:** Demographic data, basal body composition and biochemical parameters of study subjects according to adiposity

	**Normal-weight (*n* = 27)**	**Overweight (*n* = 12)**	**Obesity (*n* = 38)**	**Morbid obesity (*n* = 22)**	***p*-value**
Gender (male/female)	9/18	7/5	15/23	7/15	0.470^a^
Age (years)	35.4 ± 8.9	42.4 ± 15.0	44.3 ± 12.8^*^	44.8 ± 10.4^*^	0.005
Height (m)	1.7 ± 0.1	1.7 ± 0.1	1.6 ± 0.1	1.6 ± 0.1	0.227
Body weight (kg)	63.3 ± 11.1	79.6 ± 9.0^*^	93.2 ± 12.6^*†^	126.8 ± 18.4^*†¥^	< 0.001
BMI (kg/m^2^)	22.6 ± 2.5	28.5 ± 1.4^*^	34.9 ± 2.3^*†^	47.1 ± 5.3^*†¥^	< 0.001
FM (kg)	14.3 ± 4.9	25.1 ± 6.8^*^	37.6 ± 8.5^*†^	63.4 ± 13.1^*†¥^	< 0.001
FFM (kg)	53.2 ± 9.6	45.6 ± 10.5	46.5 ± 11.6	58.6 ± 11.9^†¥^	0.001
Haemoglobin (g/dL)	14.4 ± 1.4	15.3 ± 1.2	14.0 ± 1.4^†^	14.1 ± 1.2	0.053
Haematocrit (%)	42.4 ± 3.7	45.0 ± 3.5	41.6 ± 3.8^†^	41.4 ± 3.2^†^	0.038
RBC (× 10^6^/µL)	4.76 ± 0.4	4.9 ± 0.4	4.7 ± 0.4	4.8 ± 0.3	0.551
Iron (µg/dL)	99.9 ± 30.2	133.0 ± 30.3	73.8 ± 25.5^*†^	71.4 ± 30.9^*†^	0.001
Ferritin (ng/mL)	70.2 ± 70.8	161.0 ± 69.0	79.1 ± 100	151.0 ± 204.0	0.113
TSI (%)	26.8 ± 9.03	37.0 ± 13.9	17.9 ± 6.53^*†^	18.2 ± 8.35^*†^	< 0.001
Transferrin (mg/dL)	282.0 ± 49.1	267.0 ± 41.5	298.0 ± 40.0	287.0 ± 39.6	0.529

Data show mean ± standard deviation. *p*-value was calculated with ANOVA. ^a^ *p*-value was calculated with Chi-square. * denotes statistically significant differences (*p* < 0.05) with regard to normal-weight individuals. ^†^ denotes statistically significant differences (*p* < 0.05) with regard to overweight individuals. ^¥^ denotes statistically significant differences (*p* < 0.05) with regard to individuals with obesity. BMI: body mass index; FM: fat mass; FFM: fat-free mass; RBC: red blood cell; TSI: transferrin saturation index

**Fig. 1:**
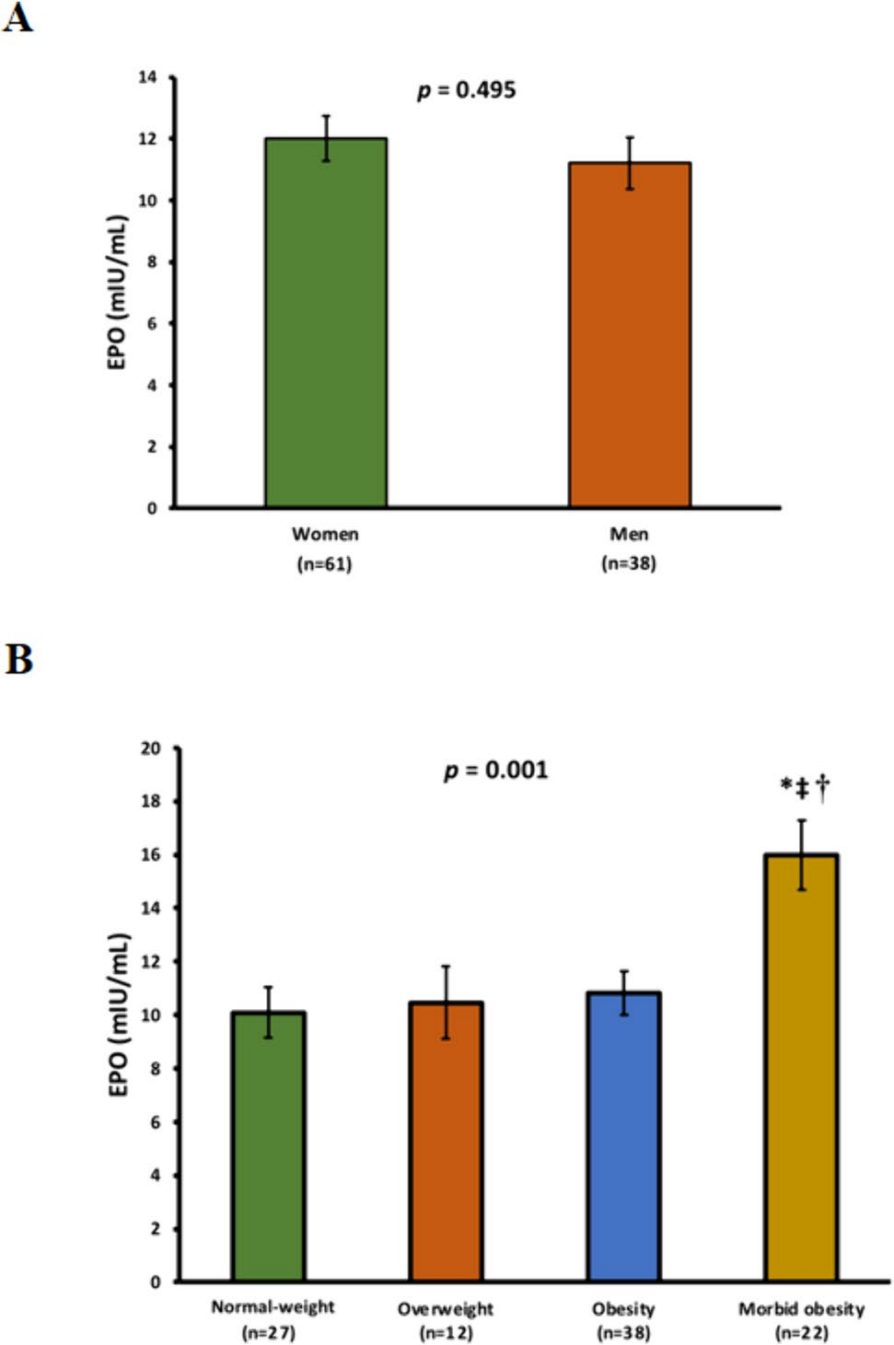
Comparison of EPO levels according to gender and adiposity**.** **A** Differences in EPO levels according to gender evaluated by Student´s *t*-test, **B** Differences among the participants according to body mass index (BMI) classified into the following categories: normal-weight (< 25 kg/m^2^), overweight (25–29.9 kg/m^2^), obesity (30–39.9 kg/m^2^) and morbid obesity (≥ 40 kg/m^2^). The data are presented as the mean (SE). * denotes statistically significant differences (*p* < 0.05) with regard to normal-weight subjects, ‡ denotes statistically significant differences (*p* < 0.05) with regard to overweight subjects, and † denotes statistically significant differences (*p* < 0.05) with regard to subjects with obesity, evaluated using analysis of covariance (ANCOVA) adjusted by age and gender

**Fig. 2:**
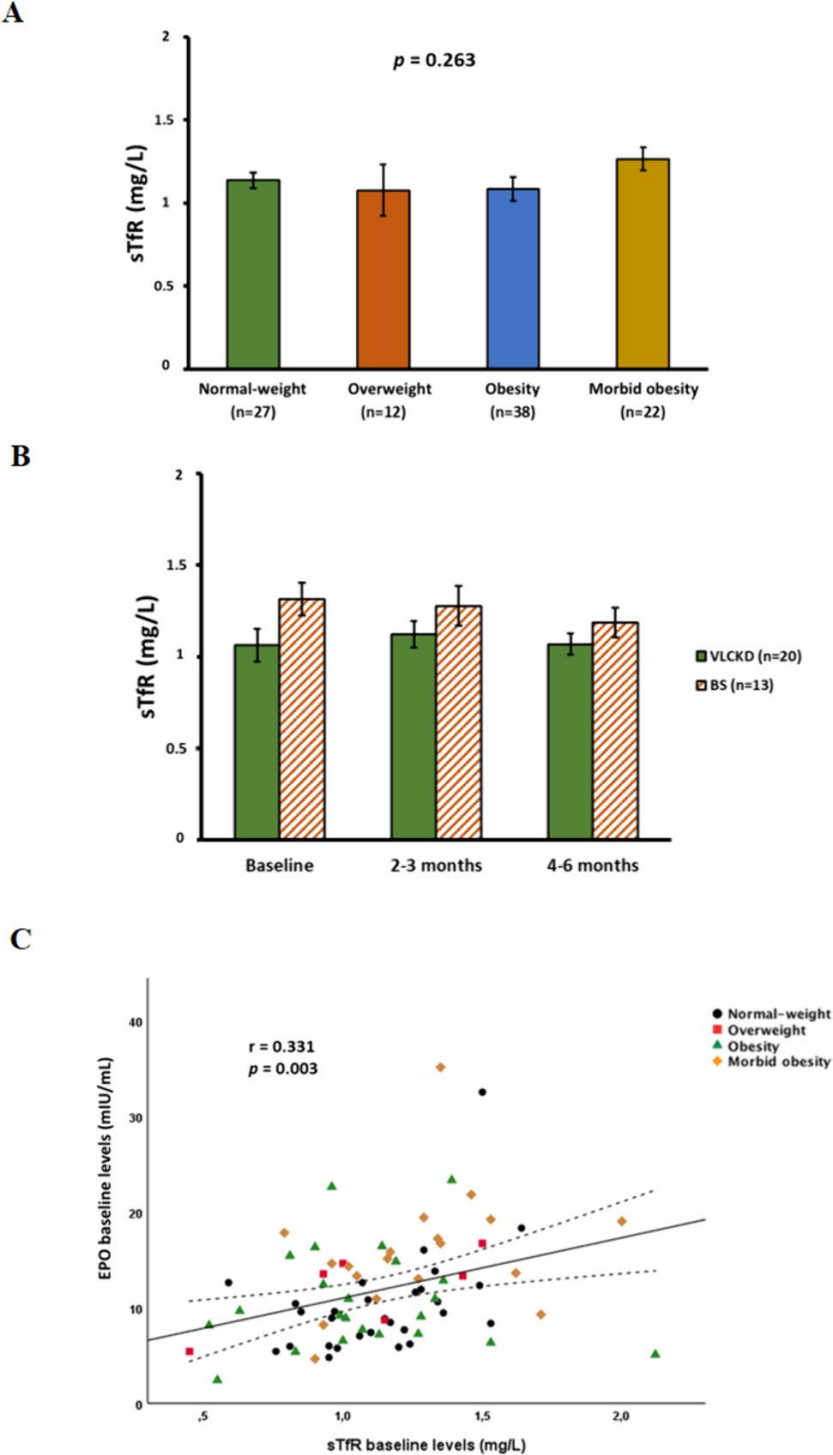
Evaluation of soluble transferrin receptor values according to gender and type of intervention and its correlation with erythropoietin levels. **A** Differences among the participants according to body mass index (BMI) classified into the following categories: normal-weight (< 25 kg/m^2^), overweight (25–29.9 kg/m^2^), obesity (30–39.9 kg/m^2^) and morbid obesity (≥ 40 kg/m^2^), evaluated using analysis of covariance (ANCOVA) adjusted by age and gender. The data are presented as the mean (SE), **B** Effect of the weight-reduction therapies on sTfR levels throughout follow-up. Data show mean differences of sTfR levels from baseline to endpoint and the end of follow-up after the very-low-calorie ketogenic diet (VLCKD) and bariatric surgery (BS) evaluated by repeated measures ANCOVA, **C** Association between EPO and sTfR. Scatterplot representing the association between EPO baseline levels and sTfR baseline levels. The centre line represents the linear regression trendline. The lines above and below the centre line represent the upper and lower bounds of the 95% confidence interval around the trendline. *r*: correlation coefficient evaluated by the Rho Spearman test; *p*: *p* value

The levels of EPO were first evaluated according to gender and excess adiposity at baseline. Regarding gender, no statistically significant differences were found between men and women (Fig. 1A). In relation to the level of adiposity, after age adjustment, EPO levels were found to be higher in the morbid obesity group (16.0 ± 6.1 mIU/mL) compared with the other groups of patients (10.8 ± 5.1 mIU/mL in the obesity group, 10.4 ± 4.7 mIU/mL in the overweight group, and 10.0 ± 5.3 mIU/mL in the normal-weight group; *p* = 0.001) (Fig. 1B). Regarding sTfR values, no significant differences were observed in relation to excess adiposity (Fig. 2A). In addition, while a slightly lower haematocrit was observed in the obesity groups, accompanied by lower iron levels and transferrin saturation index (TSI), no statistically significant differences were found regarding red blood cell (RBC) count and haemoglobin levels (Table 1).

**Fig. 3:**
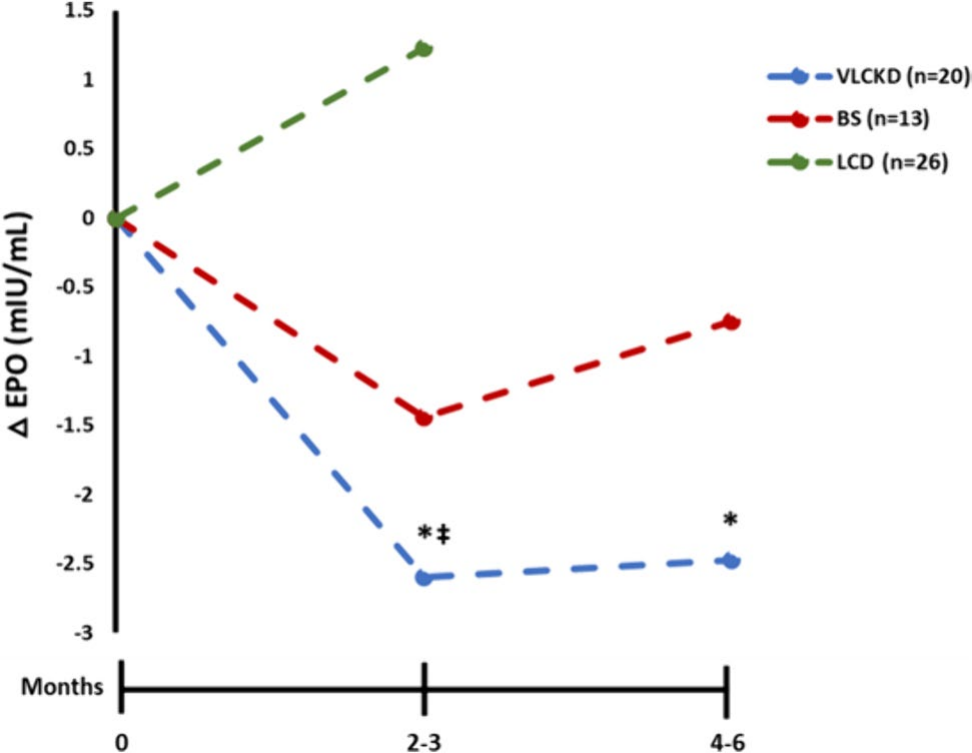
Effect of the weight-reduction therapies on EPO levels throughout follow-up. Data show mean differences of EPO levels from baseline to endpoint and the end of follow-up after the three weight-reduction interventions. * denotes statistically significant differences (*p* < 0.05) in relation to baseline levels calculated with Student’s *t*-test. ‡ denotes statistically significant differences in the changes respect to baseline (*p* < 0.05) with regard to low-calorie diet (LCD), evaluated using univariate analysis of covariance (ANCOVA) adjusted by baseline BMI. VLCKD, very-low-calorie ketogenic diet; BS, bariatric surgery

All of the 69 patients within the overweight and obesity ranges in all three intervention groups included in this study achieved a statistically significant weight loss (Table 2). Thus, in the group of 20 patients treated with VLCKD, a weight loss of 19.4 kg (−20.2%) was observed at the end of follow-up, fundamentally at the expense of FM reduction (−16.5 kg, −39.1%) with only a slight loss of fat-free mass (FFM) (−3.7 kg, −7.0%). Considering β-OHB levels, as expected in this type of intervention, a significant increase was observed at the endpoint (1.3 ± 0.1 mM) in comparison with baseline values (0.3 ± 0.02 mM), *p* < 0.001, while glucose levels progressively decreased over time. EPO levels had also decreased significantly (Table 2, Fig. 3) at the endpoint (8.39 ± 3.7 mIU/mL), maintaining this reduction at the end of the follow-up (8.5 ± 2.7 mIU/mL) in comparison with the baseline situation (11.0 ± 4.2 mIU/mL; *p* = 0.013). In addition, no differences were observed regarding sTfR values (Fig. 2B). RBC indices and iron status after the VLCKD can be seen in Table 2.

**Table 2:** Baseline characteristics and changes induced by the weight-loss treatments in body weight, body composition and biochemical parameters

Variables	VLCKD (*n* = 20)			LCD (*n* = 26)		BS (*n* = 13)		
	Baseline	Endpoint	Follow-up	Baseline	Endpoint	Baseline	Endpoint	Follow-up
	(0 months)	(2–3 months)	(4–6 months)	(0 months)	(3 months)	(0 months)	(3 months)	(6 months)
Gender (male/female)	6/12			13/13		0/13		
Body weight (kg)	96.0 ± 16.3	84.2 ± 13.0^‡^	76.6 ± 11.1*^‡† ^	89.5 ± 14.7	85.7 ± 14.1*	109.4 ± 16.4	85.9 ± 11.7^‡^	76.1 ± 12.1*^‡†^
BMI (kg/m^2^**)**	35.6 ± 4.4	31.2 ± 3.4^‡^	28.4 ± 2.7*^‡†^	33.3 ± 3.7	31.9 ± 3.5*	42.4 ± 5.4	33.4 ± 4.3^‡^	29.5 ± 4.2*^‡†^
FM (kg)	42.2 ± 9.2	35.0 ± 7.8^‡^	25.7 ± 5.8*^‡†^	31.2 ± 9.7	29.8 ± 9.8	54.1 ± 11.0	37.6 ± 7.8^‡^	29.1 ± 7.6*^‡†^
FFM (kg)	52.8 ± 10.3	48.6 ± 9.3^‡^	49.1 ± 9.7*^‡^	41.6 ± 10.9	38.0 ± 11.8	51.3 ± 7.1	44.9 ± 5.6^‡^	45.0 ± 5.9*^‡†^
EPO (mIU/mL)	11.0 ± 4.2	8.4 ± 3.7^‡^	8.51 ± 2.7*^‡^	10.1 ± 4.2	11.2 ± 5.9	16.6 ± 8.2	15.2 ± 7.5	15.9 ± 9.3
sTfR (mg/L)	1.1 ± 0.4	1.1 ± 0.3	1.1 ± 0.2	–	–	1.3 ± 0.3	1.3 ± 0.4	1.2 ± 0.3
Haemoglobin (g/dL)	14.1 ± 1.3	14.3 ± 1.2	14.0 ± 1.3*	14.6 ± 1.4	14.3 ± 1.4*	13.6 ± 1.7	12.8 ± 1.6^‡^	12.8 ± 1.2*^‡^
Haematocrit (%)	41.6 ± 3.2	41.8 ± 2.9	41.4 ± 3.1	43.2 ± 3.9	40.7 ± 8.2	40.3 ± 4.2	38.0 ± 4.1^‡^	37.9 ± 3.3*^‡^
RBC (× 10^6^/µL)	4.8 ± 0.4	4.8 ± 0.4	4.7 ± 0.4*^†^	4.8 ± 0.4	4.7 ± 0.5*	4.6 ± 0.3	4.4 ± 0.3^‡^	4.3 ± 0.3*^‡^
Iron (µg/dL)	84.3 ± 25.7	67.7 ± 18.3^‡^	75.5 ± 19.0*	–	–	74.4 ± 32.9	56.2 ± 19.2^‡^	64.5 ± 17.7*
Ferritin (ng/mL)	105.0 ± 102.9	127.0 ± 103.6^‡^	114.8 ± 81.5*	–	–	56.0 ± 43.4	75.2 ± 67.8	70.2 ± 68.3
TSI (%)	20.5 ± 6.6	19.4 ± 5.4	22.1 ± 6.3*^†^	–	–	19.2 ± 8.3	18.2 ± 8.0	19.5 ± 6.1
Transferrin (mg/dL)	295.3 ± 38.8	250.4 ± 28.7	245.4 ± 25.5	–	–	279.4 ± 39.6	232.7 ± 45.6	245.2 ± 52.9
Glucose (mg/dL)	96.8 ± 12.2	78.6 ± 9.9^‡^	77.3 ± 8.5*^‡^	–	–	–	–	–
β-OHB (mmol/L)	0.3 ± 0.02	1.3 ± 0.1^‡^	0.2 ± 0.2*^‡†^	–	–	–	–	–

Data show mean ± standard deviation. *p*-value was calculated with ANOVA. ^a^*p*-value was calculated with Chi-square. * denotes statistically significant differences (*p* < 0.05) in time course calculated with repeated measures ANOVA in very-low-calorie ketogenic diet (VLCKD) and bariatric surgery (BS), and calculated with Student’s *t*-test in low-calorie diet (LCD). ‡ denotes statistically significant differences (*p* < 0.05) in relation to baseline levels calculated with Student’s *t*-test. † denotes statistically significant differences (*p* < 0.05) in relation to endpoint levels calculated with Student’s *t*-test. BMI: body mass index; FM: fat mass; FFM: fat-free mass; EPO: erythropoietin; RBC: red blood cell; TSI: transferrin saturation index; β-OHB: β-Hydroxybutyrate

In the group of 26 patients who underwent an LCD, a statistically significant weight loss of 3.8 kg (4.2%) was also observed over 2 months. Although a slight reduction in both FM and FFM was shown in this group of subjects, no significant differences were found. Regarding the group of patients who underwent bariatric surgery, complete data throughout follow-up were available for 13 subjects. The strong body-weight loss found (−33.3 kg, −30.4%) was characterised by a statistically significant loss of FM (−25.0 kg, −46.2%) and a decrease of 6.3 kg (−12.3%) of FFM. However, no significant differences were observed in EPO levels between baseline and at endpoint or follow-up, either in the LCD group or in the bariatric surgery group (Table 2, Fig. 3), nor were differences observed regarding sTfR in the latter. No sTfR data were available regarding the LCD group (Fig. 2B). Nevertheless, a slight reduction was found in the RBC count, haemoglobin values in both groups with respect to baseline values, as well as a reduction in haematocrit and iron levels in the case of the bariatric surgery group (Table 2).

When differences from baseline were evaluated between the three interventional groups, the VLCKD show higher decrease at the endpoint respect to BS and LCD, and these differences were statistically significant even though the analysis was adjusted for baseline BMI (*p* = 0.044).

**Fig. 4:**
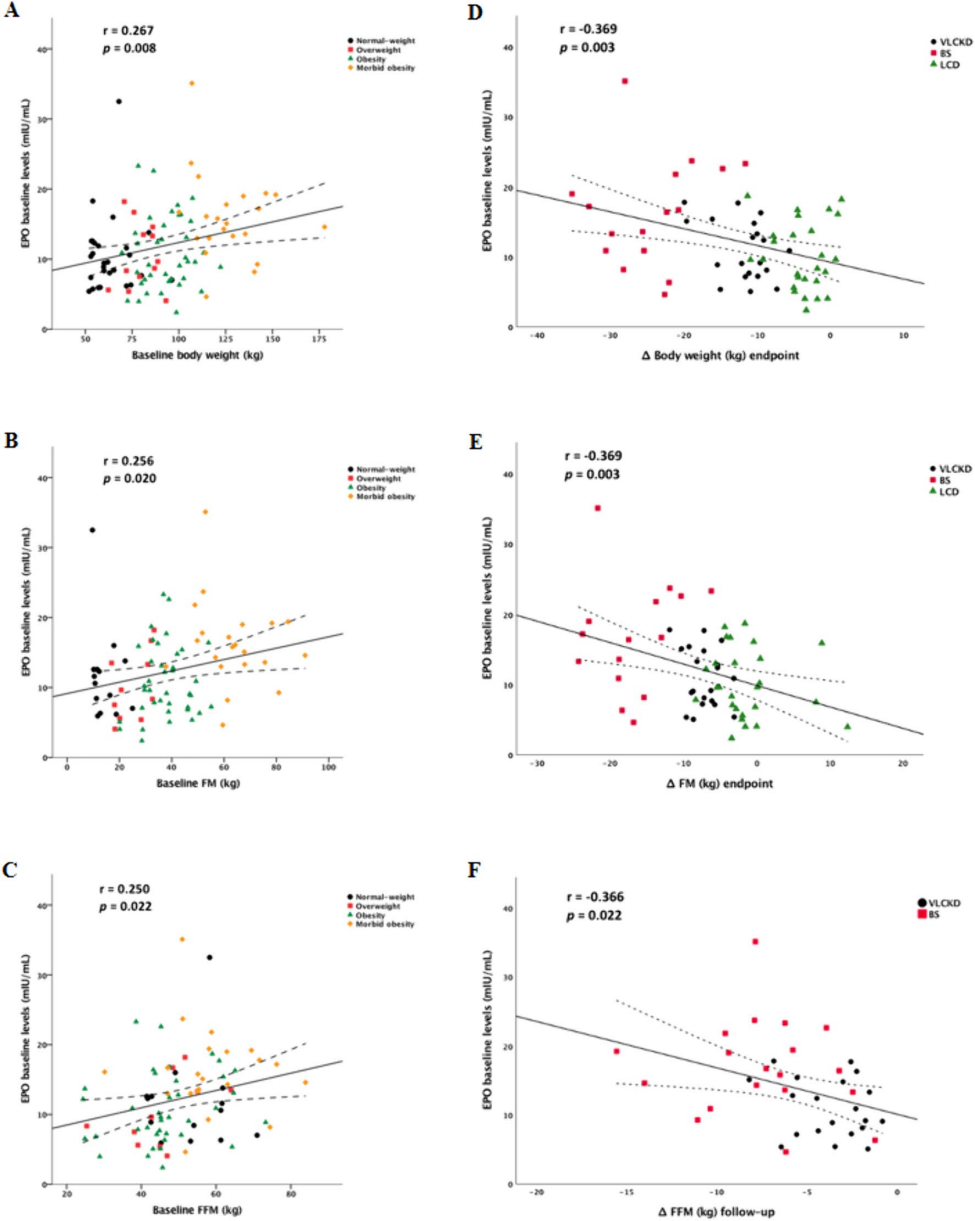
Association between baseline EPO levels and changes in body weight and body composition**.** Scatterplot representing the association between baseline EPO levels and body weight, fat mass (FM) and fat-free mass (FFM) at baseline (Fig. 4A–C) and between baseline EPO levels and changes in body weight and fat mass in the baseline-endpoint period and FFM in the endpoint-follow-up period after the weight-loss therapies (Fig. 4D–F). Each point in the plots refers to individual changes. The centre line represents the linear regression trendline. The lines above and below the centre line represent the upper and lower bounds of the 95% confidence interval around the trendline. *r*: correlation coefficient evaluated by the Rho Spearman test; *p*: *p* value; VLCKD, very-low-calorie ketogenic diet; BS, bariatric surgery; LCD, low-calorie diet

Finally, to elucidate whether the observed changes in EPO were associated with changes in body weight and body composition, correlation analyses were performed using data from all patients. Thus, at baseline (Fig. 4A–C), EPO levels were positively correlated with body weight (*r* = 0.27; *p* = 0.008), FM (*r* = 0.26; *p* = 0.020) and FFM (*r* = 0.25; *p* = 0.022). A negative correlation was also observed between EPO baseline levels and changes in body weight (*r* = −0.37; *p* = 0.003) and FM (*r* = −0.37; *p* = 0.003) in the baseline-endpoint period and in FFM (*r* = −0.37; *p* = 0.022) in the endpoint-follow-up period (Fig. 4D–F). On the other hand, although a positive correlation was found between baseline EPO levels and sTfR (*r* = 0.33; *p* = 0.003) (Fig. 2D), no associations were observed between the latter and changes in body weight or body composition. In addition, no significant correlation was found between EPO levels and β-OHB in the VLCKD group either at the endpoint or at follow-up.

## Discussion

In the present study, severe obesity was associated with increased EPO concentrations and iron dysregulation, with a positive correlation with basal weight, FM and FFM in the overall sample. After weight loss in patients with obesity, specifically that induced by a VLCKD, EPO circulating levels decreased coinciding with the moment of maximum ketosis, which was maintained over time and no effect on the EPO time-course was observed after LCD and bariatric surgery. In addition, high baseline EPO levels were correlated with higher impact on the course of weight loss and body composition changes in the first 2–3 months after the three different therapeutic weight-loss strategies. These results demonstrate that the increase in ketone bodies yielded by nutritional ketosis do not induce an increase in EPO secretion and they suggest a potential role of EPO on counteracting the metabolic stress associated to obesity and circulating levels of EPO could be a biomarker of the health status of patients under a weight-loss therapy.

In agreement with our results, EPO has previously been found to be increased in patients with obesity and anaemia [14], in subjects with metabolic syndrome and in individuals with abdominal obesity component [14, 15]. It is known that adipose tissue in patients with obesity produces an increased amount of proinflammatory cytokines, therefore contributing to the development of a low-grade systemic inflammation in these subjects [14, 33, 34]. The development of sideropenia could be a consequence of this obesity-associated low-grade inflammation, in addition to diets with low value of Index of Nutritional Quality for iron among other factors [33–35]. EPO levels have also been shown to be increased in this population, secondary to iron deficiency in its active metabolic pool [14]. However, in our population, clinical parameters of iron deficiency or anaemia were not observed, even though patients with obesity showed lower iron, TSI and haematocrit levels than normal-weight volunteers. On the other hand, growing evidence has also suggested that cellular hypoxia and reduced adipose tissue oxygenation may be an underlying trigger of adipose tissue dysfunction leading to metabolic variations associated with obesity and metabolic syndrome [15]. In this sense, hypoxia is a known stimulator of EPO production [3].

sTfR levels were also measured in the current study. Although previous studies have also shown higher sTfR values in subjects with obesity as well as in individuals with abdominal obesity component of metabolic syndrome [15, 36], no significant variations according to the degree of adiposity were observed in our population. In addition, it is known that sTfR levels may not only be increased in iron deficiency with inadequate iron supply for erythropoiesis [37] but also due to the use of erythropoiesis-stimulating agents such as EPO [38]. However, the fact that no differences were observed in these values according to adiposity degree and after weight-loss interventions, despite their positive correlation with basal EPO levels, leads us to think that the mechanisms causing the relationship between EPO and changes in weight and body composition in these patients are independent of sTfR levels.

In our study, EPO levels decreased precisely after VLCKD intervention, at the time of maximum ketosis as evidenced by β-OHB levels, and remained within that range during follow-up. The decrease in EPO levels seen after VLCKD was not observed after LCD or bariatric surgery, suggesting a potential role of the nutritional ketosis induced by the VLCKD in this effect. On the contrary, previous studies using SGLT2 inhibitors (which can also induce moderate weight loss) showed an increase in EPO levels along with an increase in haematocrit, proposing that the hyperketonaemia induced by this drug directly stimulates circulating EPO concentrations [18, 39]. The observed decrease in EPO levels in our population after VLCKD were accompanied by higher levels of β-OHB (~ 1.3 mmol/L) than is typically shown during SGLT2 inhibition (~ 0.6 mmol/L). Even higher levels of hyperketonaemia (4–5 mmol/L) were observed in a recent study, in which EPO concentrations were shown to be significantly greater after Na-3-βOHB administration in comparison with saline infusion [18]. This confirms the high complexity of the underlying mechanisms involved in EPO variations, which is why further research in this field is needed. On the other hand, apart from hyperketonaemia, another of the proposed theories is that the transfer of enhanced but less efficient oxygen-consuming active sodium reabsorption to the distal tubule results in the expression of hypoxia-inducible factors, which stimulate erythropoiesis [16]. Obesity-related adipose tissue is also characterised by a hypoxia status providing cellular mechanisms for chronic inflammation and mitochondrial dysfunction. Likewise, higher serum EPO concentrations may suggest underlying adipose tissue hypoxemia in patients with excess adiposity [15]. In this sense, the possible influence of adipose tissue-associated hypoxia and inflammation on the response of EPO to ketone bodies could also be clinically investigated by exploring the effect of VLCKD on EPO levels in a different population such as healthy competitive bodybuilders [40].

Beyond erythropoiesis, endogenous EPO protects against FM accumulation and systemic inflammation [41]. The increased EPO levels observed in obesity could suggest that EPO is secreted to counteract the metabolic deficiencies in these patients with a variable degree of systemic inflammation related with an increase in adipose tissue and cytokine production [14, 33, 34]. In this sense, it is known that many of the benefits of VLCKD in obesity are based on its ability to exert anti-inflammatory and antioxidant effects [24, 42], contrary to what occurs with bariatric surgery, which is related to metabolic stress during the weight-loss process [43]. This could also explain why EPO levels decrease and are maintained within that range after a weight-loss therapy that is capable of improving metabolic disorders and inflammation, such as VLCKD. In accordance with this proposal, the current results could also suggest, therefore, that under a healthy metabolic, hormonal and inflammatory profile, the secretion of protective proteins, such as EPO, is not needed. In addition, those patients with higher baseline EPO levels also showed a higher decrease in body weight and FM after weight-loss treatments, suggesting an additional capacity to predict response to a weight-loss therapy. Similar results were previously observed when studying other proteins with beneficial properties, such as FGF21 [43], irisin [44], betatrophin [45] and leptin [46], among others. These hormones were associated with beneficial metabolic effects and capacity to protect against obesity in preclinical models. All of them are increased in patients with obesity and their concentration in plasma decreases after a weight-loss therapy that improves the metabolic and inflammatory status of patients with obesity, as observed in EPO levels in the current study. In this regard, it has already been demonstrated that a VLCKD is able to reduce visceral FM, preserving muscle mass [22] and function [23], and that this beneficial effect on body composition was concomitant with an improvement in the inflammatory and oxidative stress status [24, 42] and a restoration of the obesity-related epigenome [47]. All of these effects were observed mainly in the first steps of this nutritional treatment when it induced a similar weight loss than that observed in patients undergoing bariatric surgery, which, as previously mentioned, is related to metabolic stress in the short-term [43]. The main difference between both weight-loss treatments regarding the effect on weight loss is the VLCKD-induced nutritional ketosis, which is the main player in the beneficial effect induced by this specific diet [24]. Not only has VLCKD also been linked to improving mitochondrial function and decreasing oxidative stress [24, 42], but also EPO promotes metabolic activity and adipocytes to increase mitochondrial function via Sirtuin 1, among other regulators of energy homeostasis [48]. Interestingly, some studies indicate that the anti-inflammatory effects of ketone bodies are actually mediated by Sirtuin 1 activity [49]. Thus, EPO variations and their metabolic effects seem to comprise a complex framework that needs to be examined in more depth to gain a greater understanding thereof.

The longitudinal design of the current study enables, for the first time, the evaluation of time-course changes in EPO levels in obesity after three different weight-loss therapies (VLCKD, LCD and bariatric surgery). The presence of a normal-weight control group also contributes to making the data more robust. Although it should be taken into account that, in the current study, the bariatric surgery group is mainly represented by women, no differences were found regarding EPO levels and gender. Moreover, the three different treatments were not matched for BMI because bariatric surgery is prescribed for patients with severe obesity. However, this issue does not invalidate the results because the effect of the three interventions were assessed respect to the baseline levels, comparing each patient with himself and after a statistical analysis adjusted for baseline BMI, differences in EPO levels between interventional groups were maintained. In addition, considering that this is an observational association study, and that, therefore, only hypotheses related to changes in erythropoiesis can be proposed, long-term prospective research is needed to examine causality.

## Conclusion

The increased EPO levels found in severe obesity in our sample, their association with basal body weight, FM and FFM and their correlation with higher weight loss and changes in body composition in the short term after weight-loss therapies, suggest the role of EPO as a contributing factor in adipose tissue energy homeostasis as well as in the possible detection of patients who are more likely to respond initially to weight-loss therapies. Contrary to what was previously described in the literature in relation to hyperketonaemia and EPO concentrations, in the present study, VLCKD-induced nutritional ketosis promoted a decrease in EPO levels, synergistically with weight loss, suggesting a potential role of nutritional ketosis in this effect. In summary, this study demonstrates that the decreased EPO levels coinciding with the moment of maximum ketosis after the VLCKD intervention could contribute to discarding the previously suggested role of ketone bodies in the stimulation of EPO concentrations. This suggests the great complexity of the underlying mechanisms involved in erythropoiesis associated to obesity. In addition, it also provides new evidence of a potential role of EPO as a marker of the metabolic status related to obesity therapies.

## Data Availability

Data are available on request to the corresponding author, only if appropriate and with the permission of the subject.
